# Non-SMC Condensin I Complex Subunit H (NCAPH) Is Associated with Lymphangiogenesis and Drug Resistance in Oral Squamous Cell Carcinoma

**DOI:** 10.3390/jcm9010072

**Published:** 2019-12-27

**Authors:** Hiroyuki Shimomura, Tomonori Sasahira, Chie Nakashima, Miyako Kurihara-Shimomura, Tadaaki Kirita

**Affiliations:** 1 Department of Molecular Pathology, Nara Medical University, Kashihara 634-8521, Japan; hiroz@naramed-u.ac.jp (H.S.); c-nakashima@naramed-u.ac.jp (C.N.); miyako@naramed-u.ac.jp (M.K.-S.); tkirita@naramed-u.ac.jp (T.K.); 2 Department of Oral and Maxillofacial Surgery, Nara Medical University, Kashihara 634-8521, Japan

**Keywords:** NCAPH, lymphangiogenesis, anticancer drug resistance, prognosis, oral cancer

## Abstract

Background: Head and neck cancer, including oral squamous cell carcinoma (OSCC), is the sixth most common malignancy. OSCC has strong invasive ability, and its malignant potential is closely associated with local expansion and lymph node metastasis. Furthermore, local or nodal recurrence worsens OSCC prognosis. In our previous cDNA microarray analysis, non-structural maintenance of chromosome (SMC) condensin I complex subunit H (*NCAPH*) was identified as an upregulated gene in recurrent OSCC. Although NCAPH has several functions in tumors, its role in OSCC is unknown. Methods: In this study, we examined NCAPH expression in OSCC and performed a functional analysis of human OSCC cells. Results: *NCAPH* expression was higher in OSCC than in normal oral mucosa. In immunohistochemistry using 142 OSCC specimens, the immunostaining of NCAPH was strongly associated with nodal metastasis and lymphatic infiltration. In multivariate analysis using the Cox proportional hazards model, NCAPH expression was an independent poor prognostic indicator for OSCC. Moreover, NCAPH promoted the migration and adhesion of endothelial cells to OSCC cells and promoted the resistance to platinum anticancer drugs. Conclusions: Our present findings suggest that NCAPH is a novel diagnostic and therapeutic target in OSCC.

## 1. Introduction

Head and neck squamous cell carcinoma (HNSCC), including oral squamous cell carcinoma (OSCC), is the sixth most common malignancy and has aggressive potential [1]. The synergic effects of tobacco smoking and alcohol consumption are the major risk factors for OSCC, and the other risk factors for oral carcinogenesis include betel quid chewing, malnutrition, poor oral hygiene, mouthwash, immunosuppression, and human papillomavirus infection associated with certain sexual behaviors [2]. OSCC is graded at the histological level. More specifically, grade I (well differentiated) includes tumors composed of <25% undifferentiated cells, grade II (modeerately differentiated) lesions with 25–50% undifferentiated cells, and grade III (poorly differentiated) lesions with >50% undifferentiated cells [3]. OSCC often causes difficulty in chewing and swallowing, produces sores that do not heal, leads to ill-fitting dentures, and results in speech and cosmetic disorders [2,4]. Given the rich blood supply and lymphatic reflux of the oral mucosa, OSCC is also prone to local invasion and nodal metastasis [2]. The 5-year survival rate of OSCC has remained at approximately 50% over the past 30 years [5]. Although surgical resection is the general rule for OSCC treatment, oral surgery also causes tissue damage and functional impairment. Therefore, elucidating the molecular mechanisms of carcinogenesis is essential for the early detection of OSCC.

Condensin is a multiprotein complex that plays a pivotal role in chromosome-wide gene regulation by controlling chromosome assembly and separation in the mitotic and meiotic cell cycles of proliferative cells [6,7,8]. Many eukaryotic cells carry two different types of condensin complexes, namely, condensins I and II, and both complexes share the same pair of structural maintenance of chromosome (SMC) 2 and 4 subunits belonging to the superfamily of chromosomal ATPases [6]. Additionally, condensin I complexes feature non-SMC condensin I complex subunits H (NCAPH), G (NCAPG), and D2 (NCAPD2) [6,7]. Previously we performed comprehensive gene expression analysis on primary and recurrent OSCC by using cDNA microarray [9]. Among the upregulated genes, NCAPH was 6.6-fold overexpressed in recurrent OSCC related to primary OSCC. Previous reports using data from The Cancer Genome Atlas (TCGA) suggested that NCAPH is upregulated in several malignancies, including HNSCC [7]. NCAPH overexpression is associated with resistance to carboplatin and radiation in ovarian cancer and colorectal cancer (CRC), respectively [10,11]. In colon cancer, NCAPH promotes tumor formation, cell proliferation, and apoptosis and G2/M arrest inhibition [8]. NCAPH is also expressed in hormone-sensitive prostate cancer and is involved in castration resistance, local progression (T factor), nodal metastasis, cell proliferation, migration, and invasion [7,12]. However, there is substantial uncertainty regarding the role of NCAPH in cancer cells. The overexpression of NCAPH is implicated in improved colon cancer prognosis [8], whereas the high expression of NCAPH is correlated with poor prostate cancer prognosis [7,12]. Furthermore, gene and protein expression and molecular function of NCAPH in OSCC are unclear. In this study, we examined the relationship between NCAPH expression and the clinicopathological characteristics of OSCC.

## 2. Materials and Methods

### 2.1. Tumor Specimens

This study was performed in accordance with the ethical standards presented in the Declaration of Helsinki and was approved by the Medical Ethics Committee of Nara Medical University, Kashihara, Japan (approval number: 719). A total of 142 formalin-fixed, paraffin-embedded (FFPE) samples from patients with OSCC (89 males; median age, 65.9 years; age range, 44–87 years) were used in this study. Among those cases used in immunohistochemistry, fresh-frozen samples from 30 patients with primary OSCC (21 males; median age, 68.8 years; age range, 56–79 years) and 10 samples of nontumor oral mucosa (7 males; median age, 45.3 years; age range, 32–62 years) were utilized for NCAPH expression analysis. All specimens were randomly selected at the Department of Oral and Maxillofacial Surgery, Nara Medical University Hospital. None of the patients had received radiotherapy and/or chemotherapy before surgical resection. Written informed consent was obtained from all patients, and medical records and prognostic follow-up data were obtained from the records of each patient in a database managed by the hospital. The follow-up period was 519–1981 days (mean, 1466 days; median, 1562 days). Tumor stage was determined according to the TNM classification system (eighth edition) of the Union for International Cancer Control, and the histological grade of OSCC was classified according to the criteria of the World Health Organization. The confirmation of histopathological diagnoses and OSCC grading was performed by a board certified pathologist (TS) and two authors (HS and TK), respectively.

### 2.2. Immunohistochemistry

Consecutive 3 μm sections were cut from each FFPE block, and immunohistochemistry was performed by using the EnVision + Dual Link System (Dako, Carpinteria, CA, USA). Antigen retrieval was performed via microwave treatment (95 °C) in citrate buffer (pH 6.0) for 20 min. After endogenous peroxidase activity was blocked via incubation with 3% H_2_O_2_–methanol for 10 min, nonspecific binding was prevented by incubation with 10% skim milk solution (Morinaga Milk, Tokyo, Japan) for 20 min. The sections were incubated for 2 h at room temperature with a primary antibody against NCAPH (Novus Biologicals, Centennial, CO, USA) diluted to 0.5 μg/mL. Each specimen was color developed with diaminobenzidine (DAB) solution (Dako) and was counterstained using Meyer’s hematoxylin (Sigma-Aldrich, St. Louis, MO, USA). The immunostaining of all samples was performed under the same antibody reaction and DAB exposure conditions.

All slides were independently examined by two observers (HS and TS) who were blinded to the individual clinicopathological parameters of the patients. Immunoreactivity was evaluated using the German immunoreactive score system, which is based on the proportional and intensity scores [12]. Proportional scores were assigned according to the percentage of immunopositive cells: 0, 0%; 1, 1–10%; 2, 11–35%; 3, 36–70%; and 4, 71–100%. Intensity scores were defined according to the immunostaining strength: 0, negative; 1, weak; 2, intermediate; and 3, strong. The total score was calculated by the sum of the proportional and intensity scores (its grades ranged from 0–7). We categorized immunoreactivity into four grades based on the total score: Grade 0, total score of 0; Grade 1, total score of 2–4; Grade 2, total score of 5 or 6; and Grade 3, total score of 7. Patients with grades 2 and 3 immunoreactivity were regarded as high expression of NCAPH [13]. Each specimen was scored in five distinct areas, and the resultant five scores were averaged and rounded to the nearest whole number [14]. The matching rate of the score between these two observers was 80.3% (114/142), and any disagreements were resolved by reaching a consensus.

### 2.3. Quantitative Reverse Transcription Polymerase Chain Reaction (qRT-PCR)

Total RNA was extracted using an RNeasy Mini Kit (Qiagen, Valencia, CA, USA), and total RNA (1 μg) was synthesized using a ReverTra Ace qRT Kit (Toyobo, Osaka, Japan). Quantitative reverse transcription polymerase chain reaction (qRT-PCR) was performed with a StepOne Plus Real-Time PCR system (Applied Biosystems, Foster City, CA, USA) using TaqMan Fast Universal PCR Master Mix (Applied Biosystems). Analysis was performed using the relative standard curve quantification method. TaqMan gene expression assays for *NCAPH* (ID: Hs01010752_m1) and glyceraldehyde-3-phosphate dehydrogenase (*GAPDH*) (ID: Hs03929097_g1) were purchased from Applied Biosystems. *NCAPH* expression levels were normalized to *GAPDH* expression levels.

### 2.4. Cell Culture

The human OSCC lines HSC3 and KON were obtained from the Health Science Research Resources Bank, National Institute of Biomedical Innovation, Osaka, Japan. Total RNA from the normal tongue was purchased from Biochain Institute (Newark, CA, USA) and was used as a control. Cells were maintained in Dulbecco’s modified Eagle medium (Wako Pure Chemical Industries, Osaka, Japan) supplemented with 10% fetal bovine serum (Nichirei Biosciences, Tokyo, Japan) in 5% CO_2_ in air at 37 °C.

### 2.5. Immunoblotting

Whole-cell lysate was obtained using M-PER mammalian protein extraction reagent (Thermo Fisher Scientific, Rockford, IL, USA), and 50 μg of the lysate was subjected to immunoblotting by using 12.5% SDS-PAGE gels, followed by electrotransfer to polyvinylidene fluoride membranes (Novus Biologicals). The membranes were incubated with anti-NCAPH antibody (Abcam) followed by peroxidase-conjugated IgG (MBL, Nagoya, Japan). The immune complex was visualized using the ECL Western Blotting Detection System (GE Healthcare, Amersham, UK). Anti-GAPDH antibody (Santa Cruz Biotechnology, CA, USA) was used as an internal control.

### 2.6. Transient Transfection

Silencer Select RNAi, which is a short interfering RNA (siRNA) for *NCAPH* (ID: s225959), was purchased from Ambion. AllStars Negative Control siRNA (Qiagen) was used as a control. siRNA (10 nM) was transfected using Lipofectamine 2000 (Invitrogen, Carlsbad, CA, USA).

### 2.7. Anticancer Resistance Assays

OSCC cells were treated with 1 μm cisplatin (Wako Pure Chemical), carboplatin (Wako Pure Chemical), or nedaplatin (Wako Pure Chemical). Resistance to anticancer drugs was monitored using a MarkerGene Multiple Drug Resistance Microtiterplate Assay Kit (Marker Gene Technologies, Eugene, OR, USA) and was measured using a SpectraMax M2 multidetection microplate reader (Molecular Devices, Sunnyvale, CA, USA) at an emission wavelength of 504 nm and an excitation wavelength of 538 nm.

### 2.8. Interaction Assays of Oral Squamous Cell Carcinomas (OSCCs) and Endothelial Cells

Primary human umbilical vein endothelial cells and primary human dermal lymphatic microvascular endothelial cells (HDLMVECs) were purchased from Cell Applications (San Diego, CA, USA) and maintained in endothelial (Cell Applications) and microvacular endothelial growth media (Cell Applications), respectively, under 5% CO_2_ in air at 37 °C. CytoSelect Tumor-Endothelium Adhesion Assay (Cell Biolabs, San Diego, CA, USA) and CytoSelect Tumor Transendothelial Migration Assay systems (Cell Biolabs) were used to test the reciprocal actions of OSCCs and endothelial cells. Adherent or migrating cells were detected using a Multiskan GO Microplate Spectrophotometer (Thermo Fisher Scientific) at 480 nm/520 nm.

### 2.9. Statistical Analysis

JMP13 (SAS Institute, Cary, NC, USA) software was used for statistical analyses. Fisher’s exact test was used to determine the significance of NCAPH expression and clinicopathological variables of OSCC. Disease-free survival (DFS) curves for patient outcomes were generated using the Kaplan–Meier method, and statistical significance was assessed using the log-rank test. To identify independent risk factors, univariate Cox regression analysis was used for all variables. Further, factors with statistical significance according to the results of the univariate Cox regression analysis were included in the multivariate Cox regression analysis (described as the hazard ratio with 95% confidence intervals [CIs] together with the *p*-value) [15]. *p* < 0.05 indicated statistical significance.

## 3. Results

### 3.1. Relationship between NCAPH Expression and Clinicopathological Factors

**Figure 1:**
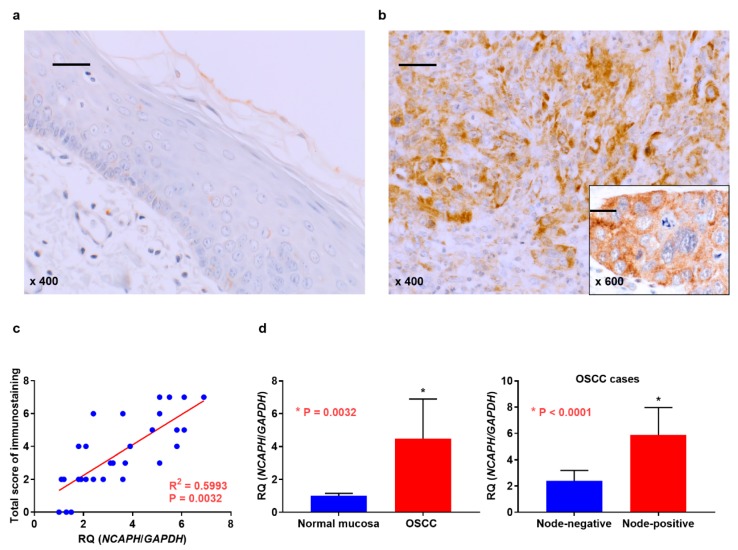
Expression analysis of NCAPH. (**a**) Weak and/or no NCAPH expression was detected in the normal oral mucosa. (**b**) NCAPH expression was observed in the cytoplasm in oral squamous cell carcinoma (OSCC). (**c**) Association of NCAPH expression with immunohistochemical grade in OSCC cases. (**d**) NCAPH expression was detected using quantitative reverse transcription polymerase chain reaction in samples of OSCC and normal oral mucosa. Differential expression of NCAPH in normal mucosa and OSCC (right) and metastatic and non-metastatic OSCC cases (left). scale bar 100 μm.

We first investigated the expression of NCAPH in 142 patients with OSCC. The location of primary OSCC was the tongue, lower gingiva, upper gingiva, oral floor, buccal mucosa, and hard palate in 52, 40, 26 11, 8, and 5 patients, respectively. The local progression of these tumors was as follows: T1 disease, 12 patients; T2 disease, 45 patients; T3 disease, 36 patients; and T4 disease, 49 patients. The clinical stage in all patients was stage I (*n* = 11), II (*n* = 29), III (*n* = 41), or IV (*n* = 61). Among all cases, 53 cases had pathology-confirmed nodal involvement. Immunoreactivity for NCAPH was negative or extremely weak in the adjacent non-cancerous oral mucosa (Figure 1a), whereas NCAPH expression was detected in the cytoplasm of OSCC cells (Figure 1b). NCAPH expression was found in 36 of 142 OSCC samples (25.4%) via immunohistochemical analysis. Table 1 summarizes the levels of NCAPH in OSCC with respect to clinicopathological features. Among the 53 patients with nodal metastasis, 20 (37.7%) exhibited high NCAPH expression, whereas only 16 (18%) of 89 patients without lymph node metastasis displayed low NCAPH expression (*p* = 0.0159). Furthermore, high NCAPH expression was observed in 13 of 28 patients (46.4%) with lymphatic invasion, whereas 20.2% (23/114) of patients without lymphatic infiltration had low NCAPH expression (*p* = 0.0071). No significant relationship was found between NCAPH immunoreactivity and other clinicopathological factors. We then verified the expression of NCAPH in OSCC via qRT-PCR. Incidentally, expression levels of NCAPH were significantly associated with immunohistochemical grade in OSCC cases (Figure 1c). Figure 1d shows that NCAPH was upregulated in OSCC tissues relative to its expression in nonneoplastic oral mucosal tissues (*p* = 0.0032). In addition, NCAPH expression was higher in node-positive cases (*n* = 18) than in cases without nodal metastasis (*n* = 12) (*p* < 0.0001). No significant differences were identified for NCAPH expression regarding T grade, clinical stage, or other parameters.

### 3.2. Prognosis of NCAPH Expression in Patients with OSCC

**Figure 2:**
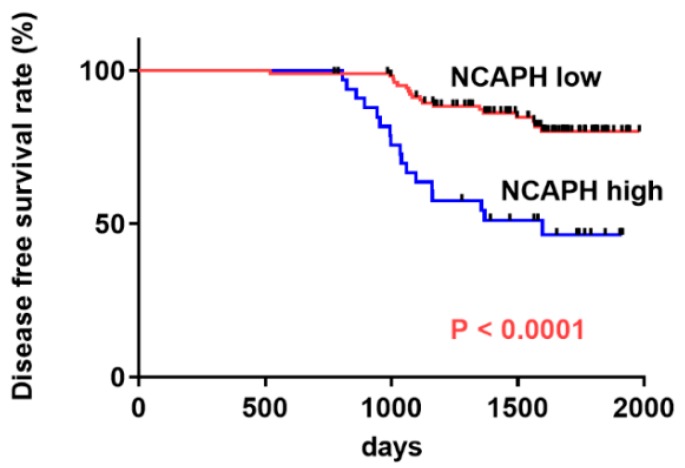
Disease-free survival (DFS) curve in patients with OSCC. The high NCAPH expression group had significantly worse DFS than the low NCAPH expression group (*p* < 0.0001).

During the follow-up period, local or metastatic recurrence occurred in 35 of 142 patients. Thereafter, we determined the association between NCAPH levels and DFS in patients with OSCC by using the Kaplan–Meier method. The DFS of patients with high NCAPH expression was remarkably shorter than that in patients with low NCAPH expression (*p* < 0.0001) (Figure 2). However, overall survival (823–3215 days, mean: 1826 days, median: 1979 days) was not associated with expression levels of NCAPH (*p* = 0.1068, data not shown). In univariate analysis, unfavorable DFS among patients with OSCC was significantly associated with clinical stage (*p* = 0.0327), nodal metastasis (*p* < 0.0001), and NCAPH expression (*p* < 0.0001). Moreover, multivariate analysis lymph node metastasis (*p* = 0.0014) and NCAPH expression (*p* = 0.0004) remained independent prognostic factors of poor OSCC prognosis (Table 2).

### 3.3. Functional Analysis of NCAPH in OSCC Cells

**Figure 3:**
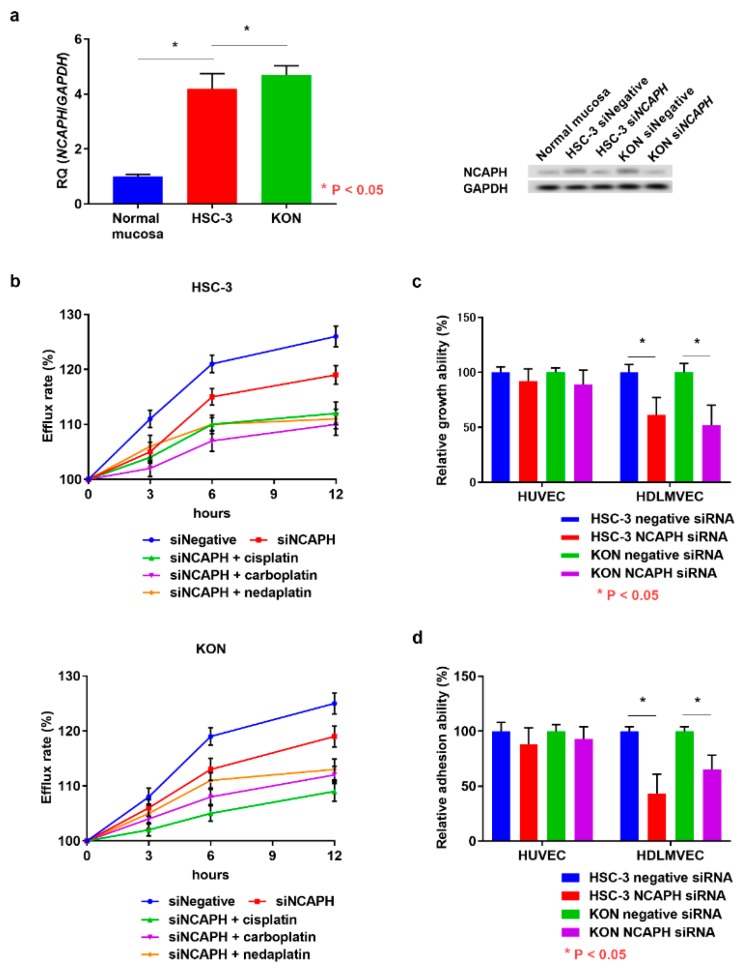
Functional analysis of NCAPH in OSCC cells. (**a**) Change in NCAPH expression in OSCC cells following treatment with *NCAPH* siRNA and negative control siRNA. (**b**) Influence of *NCAPH* siRNA treatment on anticancer drug resistance and the effects of cotreatment with *NCAPH* siRNA and cisplatin, carboplatin, or nedaplatin in OSCC cells. Effects of *NCAPH* silencing on the adhesion (**b**) and transmigration (**c**) of endothelial cells to OSCC cells. Error bar, standard deviation (SD). RQ; relative quantification.

The expression of NCAPH was higher in OSCC cells than in the normal tongue mucosa, and its expression was downregulated by NCAPH siRNA treatment (Figure 3a). Considering that NCAPH expression is related to carboplatin resistance in ovarian cancer cells, we analyzed the effect of NCAPH on OSCC cell tolerance to platinum anticancer drugs by using a multidrug-resistance (MDR) assay kit. This kit allows the measurement of the efflux of a fluorescent dye that binds to cell surface ATP-binding cassette (ABC) transporters. We have already confirmed that OSCC cell lines overexpress the ATP Binding Cassette Subfamily G Member 2 (ABCG2) and ATP Binding Cassette Subfamily B Member 1 (ABCB1) [14]. OSCC cell resistance to platinum anticancer drugs was recovered by NCAPH silencing (Figure 3b). Co-treatment with siRNA and cisplatin, carboplatin, and nedaplatin also decreased drug resistance in OSCC cells in a time-dependent manner. Given that we observed that NCAPH expression was correlated with lymphatic invasion in OSCC specimens, finally we investigated the possibility that NCAPH induces lymphangiogenesis in OSCC cells. Following incubation with NCAPH siRNA, OSCC cells displayed decreased ability to adhere to HDLMVECs (Figure 3c). Moreover, the transmigration ability of HDLMVECs toward OSCC cells was inhibited by NCAPH silencing (Figure 3d). However, NCAPH silencing had no effect on the angiogenic potential of NCAPH in OSCC cells (Figure 3c,d). These results suggest that NCAPH is a tumor-promoting factor in OSCC.

## 4. Discussion

The analysis of differential global gene expression will be useful for discovering novel markers for tumors. Previously, we identified *NCAPH* as an upregulated gene in recurrent OSCC via comprehensive expression analysis by using a cDNA microarray [9]. *NCAPH* expression is upregulated in HNSCC, gastrointestinal cancer, hepatocellular carcinoma (HCC), lung cancer, breast cancer, prostate cancer, and renal cell carcinoma compared with its levels in the normal mucosa according to TCGA data analysis [7]. Moreover, NCAPH induces tumor promotion in several malignancies [7,8,10,11,12]. In the current research, NCAPH expression was higher in OSCC than in the normal oral mucosa, and the protein was strongly involved in lymphatic invasion and nodal metastasis. In vitro studies revealed that NCAPH regulates resistance to platinum antitumor agents and lymphangiogenesis in OSCC cells. Although NCAPH expression was also an independent poor prognosticator for patients with OSCC, other reports indicated that NCAPH overexpression is strongly correlated with worse prognoses in prostate cancer [7,12]. Large-scale studies are expected in the future. NCAPH, NCAPG, and NCAPD2 are non-SMC subunits in condensin I [6,7]. Previous studies reported that NCAPG is associated with the proliferation and migration of HCC cells [16] and poor prognoses in castration-resistant prostate [17] and liver cancers [18]. Furthermore, bioinformatics analyses revealed the involvement of NCAPH in regulating cell division, mitotic nuclear division, G2/M and G1/S transitions of the mitotic cell cycle, and cell proliferation by interacting with NCAPD2, NCAPG, SMC2, SMC4, Aurora kinase A (AURKA), AURKB, cyclin-dependent kinase 1, H2A histone family member Z (H2AFZ), POC1 centriolar protein A (POC1A), and histone cluster 2 H2A family member C in prostate cancer [7]. Previously we confirmed that NCAPH interacts with NCAPG, H2AFZ, and POC1A (data not shown). Therefore, condensin I complexes, particularly NCAPH and NCAPG, might be useful diagnostic and therapeutic targets of OSCC. However, the role of NCAPD2 in cancer is poorly understood, and further analysis is needed. Generally, cisplatin-based therapy combined with other anticancer agents is the first-line chemotherapy for OSCC [19], but MDR to anticancer drugs is a major problem in OSCC treatment. MDR is characterized by the upregulation of ABC transporters that transport anticancer agents out of the cell and confer tumor cell resistance to those drugs [14]. In the current research, resistance to cisplatin, carboplatin, and nedaplatin was enhanced by NCAPH in OSCC cells. Furthermore, platinum anticancer drug therapy combined with *NCAPH* silencing decreased MDR. Conversely, we did not find any association between NCAPH and resistance to paclitaxel, docetaxel, and 5-fluorouracil (data not shown). Recent research has suggested that extracellular vesicles such as exosomes are involved in drug resistance of OSCC cells [20,21]. NCAPH may induce MDR in oral cancer cells by modulating exosome formation. However, uncertainty still remains regarding the relationship between NCAPH and resistance to treatment. Although cetuximab, which is an anti-epidermal growth factor receptor–specific chimeric monoclonal antibody, and nivolumab, which is an antibody inhibitor of programmed cell death-1 receptor, are used for molecular-targeted therapy in patients with OSCC [4], the link between these drugs and NCAPH remains unknown. A prior report indicated that patients with CRC and high NCAPH expression have better prognoses because of their increased sensitivity to chemotherapy and/or irradiation [8], whereas *NCAPH* was identified as a potential candidate radioresistance gene in CRC [11]. The associations between NCAPH and other anticancer drugs, heavy ion radiotherapy, and hyperthermia also remain unclear. Further investigations are needed to clarify the relationship between NCAPH and resistance to treatment in malignancies. Lymphangiogenesis, which is defined as the generation of new lymphatic vessels, requires the coordination of complex cellular events, including proliferation, sprouting, migration, and tube formation. In cancer, lymphangiogenesis is critical to the progression and nodal metastasis of tumor cells [22,23]. Angiogenesis and lymphangiogenesis are easily distinguished by immunostaining for CD34 and D2-40, respectively [22]. The proliferation and migration of lymphatic endothelial cells (LECs) lead to the germination and elongation of lymphatic vessels, and LECs play an important role in the interplays of cancer cells with lymphatics [23]. We previously revealed that LVD is strongly associated with tumor progression, nodal metastasis, and poor outcomes in patients with OSCC [22]. The resulting increase in LVD contributes to the formation of the metastatic niche in nodes [23]. The major tumor angiogenic and lymphangiogenic signaling pathways are typically the VEGF-A and VEGFR2 and VEGF-C/VEGF-D and VEGF receptor 3 axes, rerspectively [23,24]. Furthermore, VEGF-A [22], PDGFB [25], and prospero-related homeobox protein 1 [26] are also induced during lymphangiogenesis in OSCC. In our present studies, NCAPH promoted the transendothelial migration and adhesion of LECs to OSCC cells. According to the lymphatic-dependent sequential model of distant metastasis, tumor cells migrate to regional lymph nodes via the lymphatic system and enter the bloodstream via a remodeled vasculature in nodes or the subclavian vein via the thoracic duct; they then metastasize to distant organs [23,24,27,28]. The lymphatic microenvironment formed in lymph nodes promotes the survival of metastatic cancer cells and tumor cells that are resistant to chemotherapy [29,30]. The inhibition of NCAPH signaling might be a useful strategy for anti-lymphangiogenic therapy and MDR in OSCC. In conclusion, our results demonstrate that NCAPH is correlated with nodal metastasis via the activation of lymphangiogenesis in OSCC. NCAPH is also strongly related to poor prognosis and MDR. However, the exact mechanism underlying NCAPH-mediated signaling and functions remains unclear. It is expected that appropriate in vitro and in vivo research will be helpful in further clarifying this mechanism. Our results suggest that NCAPH could be a useful diagnostic and therapeutic target for human OSCC.

## Figures and Tables

**Table 1:** Relationship between NCAPH expression and clinicopathological parameters.

Parameters	NCAPH Expression		*p* Value **
	Low (%)	High (%)	
Gender			
Male	62 (69.7)	27 (30.3)	
Female	44 (83)	9 (17)	0.1099
Age			
≤65	74 (76.3)	23 (23.7)	
>65	32 (71.1)	13 (28.9)	0.5380
Site			
Tongue	35 (67.3)	17 (32.3)	
Other	71 (78.9)	19 (21.1)	0.1612
Smoking			
No	43 (81.1)	10 (18.9)	
Yes	63 (70.8)	26 (29.2)	0.2315
Alcohol drinking			
No	30 (69.8)	13 (30.2)	
Yes	76(76.8)	23 (23.2)	0.4055
Histological differentiation *			
Well	65 (79.3)	17 (20.7)	
Moderately, Poorly	41 (68.3)	19 (31.7)	0.1724
T classification			
T1-T3	73 (78.5)	20 (21.5)	
T4	33 (67.3)	16 (32.7)	0.1601
Clinical stage			
I-II	64 (79)	17 (21)	
IV	42 (68.9)	19 (31.1)	0.1787
Nodal metastasis			
Negative	73 (82)	16 (18)	
Positive	33 (62.3)	20 (37.7)	0.0159
Vascular infiltration			
Negative	96 (77.4)	28 (22.6)	
Positive	10 (55.6)	8 (44.4)	0.0775
Lymphovascular infiltration			
Negative	91 (79.8)	23 (20.2)	
Positive	15 (53.6)	13 (46.4)	0.0071
Perineurial invasion			
Negative	84 (75.7)	27 (24.3)	
Positive	22 (71)	9 (29)	0.6426

Relationship between expression of NCAPH and parameters were calculated by Fisher’s exact test. T classification and clinical stage were classified according to the TNM classification. * Histological differentiation: Well, well-differentiated squamous cell carcinoma; Modrately, moderately differentiated squamous cell carcinoma; Poorly, poorly differentiated squamous cell carcinoma. ** *p* value < 0.05 was regarded as statistically significant.

**Table 2:** Univariate and multivariate analysis of disease free survival.

Parameters	Univariate Analysis			Multivariate Analysis		
	HR	95% CI	*p* Value	HR	95% CI	*p* Value
Gender						
F	1.00					
M	1.1545	0.5742–2.2509	0.6789			
Age						
≤65	1.00					
>65	1.7479	0.8311–4.1209	0.1463			
Site						
Tongue	1.00					
Other	1.2126	0.6029–2.3651	0.5794			
Smoking						
No	1.00					
Yes	1.2773	0.6471–2.6545	0.4871			
Alcohol drinking						
No	1.00					
Yes	1.1418	0.5854–2.2966	0.7000			
Histology						
Well	1.00					
Mod, Por	1.3509	0.6895–2.7571	0.3849			
T factor						
T1-3	1.00					
T4	1.5607	0.7853–3.0355	0.1992			
Clinical stage						
I-III	1.00			1.00		
IV	2.0660	1.0616–4.1082	0.0327	1.4651	0.7411–2.9566	0.2725
Nodal metastasis						
Negative	1.00			1.00		
Positive	4.6133	2.3124–9.8087	<0.0001	3.1957	1.5525–6.9835	0.0014
Vascular infiltration						
Negative	1.00					
Positive	1.8162	0.7299–3.9322	0.1846			
Lymphovascular infiltration						
Negative	1.00					
Positive	1.5449	0.6836–3.1775	0.2789			
Perineural invasion						
Negative	1.00					
Positive	1.8015	0.7629–5.2882	0.1923			
NCAPH expression						
Low	1.00			1.00		
High	4.8943	2.5130–9.6537	<0.0001	3.4826	1.7524–7.0167	0.0004

Univariate and multivariate analysis was calculated by means of Cox proportional hazard model. HR and 95% CI mean hazard ratio and 95% confidence intervals, respectively.
